# *Coxiella burnetii* Employs the Dot/Icm Type IV Secretion System to Modulate Host NF-κB/RelA Activation

**DOI:** 10.3389/fcimb.2016.00188

**Published:** 2016-12-19

**Authors:** Saugata Mahapatra, Brandi Gallaher, Sydni Caet Smith, Joseph G. Graham, Daniel E. Voth, Edward I. Shaw

**Affiliations:** ^1^Department of Microbiology and Molecular genetics, Oklahoma State UniversityStillwater, OK, USA; ^2^Department of Microbiology and Immunology, University of Arkansas for Medical Sciences (UAMS)Little Rock, AR, USA

**Keywords:** *Coxiella burnetii*, NF-κB, type four secretion system, obligate intracellular, Q fever

## Abstract

*Coxiella burnetii* is the causative agent of Q fever and an obligate intracellular pathogen in nature that survives and grows in a parasitophorous vacuole (PV) within eukaryotic host cells. *C. burnetii* promotes intracellular survival by subverting apoptotic and pro-inflammatory signaling pathways that are typically regulated by nuclear transcription factor-κB (NF-κB). We and others have demonstrated that *C. burnetii* NMII proteins inhibit expression of pro-inflammatory cytokines and induce expression of anti-apoptotic genes during infection. Here, we demonstrate that *C. burnetii* promotes intracellular survival by modulating NF-κB subunit p65 (RelA) phosphorylation, and thus activation, in a Type Four B Secretion System (T4BSS)-dependent manner. Immunoblot analysis of RelA phosphorylated at serine-536 demonstrated that *C. burnetii* increases NF-κB activation via the canonical pathway. However, RelA phosphorylation levels were even higher in infected cells where bacterial protein or mRNA synthesis was inhibited. Importantly, we demonstrate that inhibition of RelA phosphorylation impairs PV formation and *C. burnetii* growth. We found that a T4BSS-defective mutant (CbΔ*dot*A) elicited phosphorylated RelA levels similar to those of wild type *C. burnetii* infection treated with Chloramphenicol. Moreover, cells infected with CbΔ*dot*A or wild type *C. burnetii* treated with Chloramphenicol showed similar levels of GFP-RelA nuclear localization, and significantly increased localization compared to wild type *C. burnetii* infection. These data indicate that without *de novo* protein synthesis and a functional T4BSS, *C. burnetii* is unable to modulate NF-κB activation, which is crucial for optimal intracellular growth.

## Introduction

*Coxiella burnetii* is a Gram-negative intracellular bacterial pathogen and the causative agent of Q fever in humans (Maurin and Raoult, 1999; Miller et al., 2006). Acute Q fever typically manifests as a self-limiting flu-like illness, with symptoms ranging from sub-clinical to debilitating, and can be fatal (Maurin and Raoult, 1999; Miller et al., 2006; Toman et al., 2012). Common chronic sequelae include endocarditis, hepatitis, and a chronic fatigue syndrome (Maurin and Raoult, 1999; Miller et al., 2006; Toman et al., 2012). Several recent Q fever outbreaks around the world exhibit the organism's global reach (Karakousis et al., 2006; Enserink, 2010; Dijkstra et al., 2012; Toman et al., 2012; Wielders et al., 2015). *C. burnetii* is environmentally stable, acquired through aerosolization, has a low infectious dose (Moos and Hackstadt, 1987), and is classified as a category B select agent (Maurin and Raoult, 1999; McQuiston and Childs, 2002; McQuiston et al., 2002; Miller et al., 2006; Toman et al., 2012). Upon inhalation, *C. burnetii* infects alveolar macrophages and replicates within a host cell-derived parasitophorous vacuole (PV) that retains many characteristics of phagolysosomes (Heinzen et al., 1999; Voth and Heinzen, 2007, 2009; van Schaik et al., 2013). The *C. burnetii* infectious cycle is ~6 days long (Coleman et al., 2004) and is highlighted by (i) invasion of the host cell by the environmentally stable Small Cell Variant (SCV) form of the bacterium, (ii) development of an acidified PV (pH < 5), (iii) differentiation of *C. burnetii* SCVs into replicative Large Cell Variants (LCVs), (iv) PV enlargement and logarithmic pathogen growth, (v) asynchronous LCV to SCV differentiation beginning around 6 days post infection (dpi), and (vi) eventual cell exit (Heinzen et al., 1999; Coleman et al., 2004; Voth and Heinzen, 2007).

Intracellular bacteria including *C. burnetii* promote infection by targeting and modulating multiple host cell molecular processes (Bhavsar et al., 2007; Böhme and Rudel, 2009; Voth and Heinzen, 2009; Lamkanfi and Dixit, 2010; van Schaik et al., 2013). Manipulation of host nuclear transcription factor NF-κB signaling is a common strategy used by microbial pathogens to thwart host innate and adaptive immune responses (Bhavsar et al., 2007; Rahman and McFadden, 2011). NF-κB is a vital regulator of genes involved in pro-inflammatory immune response, cell proliferation, and apoptosis (Beinke and Ley, 2004; Bonizzi and Karin, 2004; Hoffmann and Baltimore, 2006; Perkins, 2007; Rahman and McFadden, 2011). During normal cell function, NF-κB transcription factors—p50 (NF-κB1), p52 (NF-κB2), p65 (RelA), cRel, and RelB remain in the cytoplasm bound to the IκB inhibitory protein and are activated either via canonical or non-canonical signaling pathways (Beinke and Ley, 2004; Bonizzi and Karin, 2004; Hoffmann and Baltimore, 2006; Perkins, 2007; Rahman and McFadden, 2011). In either case, NF-κB activation and nuclear accumulation leads to inflammatory and immunomodulatory responses (Beinke and Ley, 2004; Bonizzi and Karin, 2004; Hoffmann and Baltimore, 2006; Perkins, 2007; Rahman and McFadden, 2011). NF-κB transcriptional factors regulate expression of hundreds of genes linked to the innate and adaptive immune response as well as diverse cellular processes such as proliferation, differentiation, and death (Beinke and Ley, 2004; Bonizzi and Karin, 2004; Hoffmann and Baltimore, 2006; Perkins, 2007; Rahman and McFadden, 2011). *C. burnetii* affects multiple host cell pathways regulated by NF-κB (Voth et al., 2007; Mahapatra et al., 2010). We previously demonstrated that *de novo C. burnetii* protein synthesis modulates expression of a subset of NF-κB-regulated inflammatory cytokine genes (*IL8, CCL2, CXCL1*, and *SPP1*) (Mahapatra et al., 2010). *C. burnetii* protein(s) actively reduces the RNA level of these genes relative to those found in cells containing bacteria transiently inhibited with Chloramphenicol (Cm) (Mahapatra et al., 2010). Interestingly, even though the host innate immune system is unable to contain primary *C. burnetii* infection, NF-κB-dependent cytokine production is commonly reported (Raoult and Marrie, 1995; Meghari et al., 2005, 2006, 2008; Soraya Meghari et al., 2006; Benoit et al., 2008). For example, *C. burnetii* stimulates an atypical M2 form of activation (Benoit et al., 2008). M2 cells typically have an IL-12^low^, IL-23^low^, IL-10^high^ phenotype with a variable capacity to produce inflammatory chemotactic cytokines (Benoit et al., 2008). Other prominent NF-κB-dependent cytokines induced by *C. burnetii* include TNF-α, IL-1β, IFN-γ, RANTES, MCP-1, SCYA3, SCYA4, and IL-8 (Raoult and Marrie, 1995; Ren et al., 2003; Meghari et al., 2005, 2006, 2008; Soraya Meghari et al., 2006; Benoit et al., 2008; Mahapatra et al., 2010; Amara et al., 2012; Schoffelen et al., 2014; Graham et al., 2016). In addition, *C. burnetii* regulates host apoptosis at the transcriptional level by altering expression of NF-κB-mediated cell survival-related genes (*cIAP2* and *a1/bfl-1*) (Voth et al., 2007). Moreover, avirulent *C. burnetii* activates Toll like receptor 2 (TLR-2) that typically signals via the NF-κB pathway (Zamboni et al., 2004). Therefore, existing data clearly suggest that NF-κB-dependent host cell processes are targeted by *C. burnetii*. However, essentially nothing is known about how *C. burnetii* modulates NF-κB signaling or its importance to the pathogen for intracellular survival and replication.

Intracellular bacterial pathogens including *C. burnetii* manipulate eukaryotic cell functions by secreting bacterial proteins, or effectors, that interact with host cell factors to promote intracellular survival (Voth and Heinzen, 2009; Voth et al., 2009, 2011; Toman et al., 2012; van Schaik et al., 2013; Newton et al., 2014). *C. burnetii* produces a T4BSS, and bacterially-derived virulence determinants are delivered to the host cytosol via this machinery throughout infection (Voth and Heinzen, 2009; Chen et al., 2010; Toman et al., 2012; van Schaik et al., 2013). Employing bioinformatics, bacterial two-hybrid approaches, yeast screens, and genetic screens, approximately 120 putative *C. burnetii* T4BSS effectors have been identified (Voth et al., 2009; Chen et al., 2010; van Schaik et al., 2013; Newton et al., 2014). However, it remains unknown whether the *C. burnetii* T4BSS modulates NF-κB signaling. Many immune responses seen in *in vitro* and *in vivo C. burnetii* studies have been attributed to LPS and intrinsic properties of the bacterium (Honstettre et al., 2004; Zamboni et al., 2004; Shannon et al., 2005a,b; Andoh et al., 2007; Zhang et al., 2007; Lu et al., 2008). These approaches have not addressed the possibility that *C. burnetii* actively modulates the NF-κB-mediated immune response at the cellular level using the T4BSS. We hypothesized that *C. burnetii* modulates host NF-κB signaling via the T4BSS to promote intracellular survival. In this study, we analyzed *C. burnetii*-mediated temporal modulation of NF-κB activation/signaling throughout the infectious cycle. We also identified the NF-κB signaling pathway that is activated/modulated during *C. burnetii* infection. In addition, we analyzed the effect of inhibiting NF-κB activation on *C. burnetii* growth and development. Finally, using a T4BSS-defective mutant and transient inhibition of bacterial protein synthesis, we investigated whether *C. burnetii* protein(s) activate NF-κB in a T4BSS-dependent manner.

## Materials and methods

### Growth of *C. burnetii*, tissue culture, and infection

*C. burnetii Nine* mile phase II strain stocks were cultivated in African green monkey kidney Vero cells (CCL-81; ATCC, Manassas, VA) and purified as previously described (Morgan et al., 2010). The *C. burnetii* T4BSS ΔdotA mutant strain (Beare et al., 2012) and *C. burnetii* T4BSS ΔdotA mutant complemented strain were generously provided by Dr. Bob Heinzen (NIAID Rocky Mountain Laboratories, Montana) and grown as previously described (Beare et al., 2012). Non-adherent human monocytic leukemia derived THP-1 cells (TIB-202; ATCC) were grown in 75-cm^2^ tissue culture flasks in RPMI 1640 medium (Gibco, Carlsbad, CA) supplemented with 1 mM sodium pyruvate, and 10% fetal bovine serum (FBS) at 37°C in 5% CO2 (Mahapatra et al., 2010). Hela 229 epithelial cells (CCL-1.2; ATCC) were grown in RPMI 1640 supplemented with 10% FBS and gentamicin (Invitrogen) at 37°C in 5% CO_2_ and 95% humidified air. Infections of THP-1 cells with *C. burnetii* NMII were initiated in 24-well tissue culture plates at a multiplicity of infection (MOI) of 25. Bacteria were added to 2 × 10^6^ THP-1 cells per well and incubated at 37°C for 4 h to allow close host cell-bacteria contact. Fresh media was then added to each well for a final concentration of 10^6^ cells/ml. This time point represents *T* = 0 for infectious studies.

### NF-κB modulation assay

To assay *C. burnetii* modulation of NF-κB activation, uninfected or *C. burnetii*-infected THP-1 cells were incubated for 72 hpi. Cells were then incubated in media with (+) or without (−) bacteriostatic levels (10 μg/ml) of Cm for an additional 24 h (Mahapatra et al., 2010). Total protein lysates were collected from cell pellets using laemmli sample buffer (Bio-Rad, Hercules, CA) containing protease and phosphatase inhibitor cocktails (Sigma). An NF-κB activation control was generated by treating uninfected THP-1 cells with 50 ng/ml of recombinant human TNF-α (R&D systems) for 8 h prior to total cellular protein collection (Voth et al., 2007). For temporal analysis, protein lysates from paired infected and uninfected THP-1 cells were collected at 0, 24, 48, 72, 96, and 120 hpi, with one experimental set transiently treated with 10 μg/ml of Cm for the final 24 h while the other set was mock-treated. Cell culture media was exchanged daily using centrifugation to harvest the cells and removal of the spent media followed by suspension of the cells in fresh media ± Cm. Infected and uninfected cells were handled identically and a minimum of three experiments (*N* = 3) were carried out for each time point and condition. Temporal analysis of infected and uninfected differentiated THP-1 cells treated with Rifampin to inhibit mRNA synthesis was performed by treating cells with phorbol 12-myristate 13-acetate (PMA; 200 nM; EMD Biosciences, San Diego, CA) overnight. Cells were infected with *C. burnetii*, and Rifampin (10 μg/ml) was added (+Rif) or not (−Rif) at the time of infection. Total protein lysates were collected at 2, 24, 48, 72, and 96 hpi for immunoblot analysis as described below.

### RelA inhibition

An inhibitory peptide (IP)—DRQIKIWFQNRRMKWKKNGLLSGDEDFSS (Novus Biologicals)—that competitively inhibits phosphorylation of RelA (S529/S536) and a custom synthesized control peptide (CP)—RMDRKWKQIFQNKIWRKSSDELLNDFGGD (Thermofisher scientific)—were used to determine if inhibition of NF-κB activation alters *C. burnetii* survival/growth in host cells. Initially, the IP's ability to suppress NF-κB activation in uninfected THP-1 cells was determined. Briefly, 150 μm IP or CP was added to 10^6^ THP-1 cells and incubated at 37°C, 5% CO_2_ for 4 h. Recombinant human TNF-α (200 ng/ml; R&D systems) was then added to induce NF-κB activation. Controls included untreated THP-1 cells, TNF-α only treated cells, IP only treated cells, and CP only treated cells. After an additional 12 h incubation, cells were pelleted and total protein lysates collected. After confirmation via immunoblotting that the IP does inhibit NF-κB activation, it was used to determine the requirement of NF-κB activation for *C. burnetii* survival/growth. Briefly, 10^6^ THP-1 cells treated with either 10 μg/ml of Cm (Sigma), 200 ng/ml of TNF-α (R&D systems), or IP (150 μm) were inoculated with purified *C. burnetii* NMII at an MOI of 25. Prior to inoculation, cells were treated with IP for 4 h or TNF-α for 1 h. To ensure consistent cellular response, fresh TNF-α was added every 12 h and IP every 24 h, respectively. At 72 hpi, cells were harvested for (i) total protein lysates, (ii) IFA after cytospin as previously described (Mahapatra et al., 2010) to detect PV formation, or (iii) *C. burnetii* infectious unit enumeration by sonication lysis of infected cells, serial dilution, and infection of HeLa cell monolayers followed by Fluorescent Forming Unit (FFU) calculation as described (Coleman et al., 2004; Omsland et al., 2009). Briefly, IFA was used to visualize infected cells using a Nikon Eclipse TE 2000-S fluorescence microscope. Fifteen randomly chosen fields of view (FOV) visualized at 20x magnification were counted per well to determine the average number of PVs per FOV. For FFU/ml determination, the FOV average times the area FOV/well and inoculum volume (100 ul) were multiplied by the dilution factor to determine infectious particles from each sample treatment.

### Immunoblot blot analysis

Cell lysates were separated by 12% SDS-PAGE and transferred to a nitrocellulose membrane (Pierce, Rockford, IL). The membranes were blocked for 1 h at room temperature with 5% nonfat milk in Tris-buffered saline (150 mM NaCl, 100 mM Tris-HCl, pH 7.6) containing 0.1% Tween-20 (TBST) (Voth et al., 2007). Following blocking, membranes were incubated overnight at 4°C with 5% nonfat milk in TBST having primary antibodies for the various target proteins (see following). Detection of NF-κB was performed using a rabbit monoclonal anti-human primary antibody specific to the phosphorylated Serine 536 form of RelA (Cell Signaling Technology, Danvers, MA). Activation of the non-canonical NF-κB pathway was assayed using rabbit anti-human polyclonal antibody against p100 (the precursor), and p52 (active form of NF-kappaB2, Cell Signaling Technology, Danvers, MA). Determination of *C. burnetii* density within infected THP-1 cell lysates was performed using rabbit polyclonal antibody against the *C. burnetii* 27-kDa outer membrane protein Com1 as previously described (Morgan et al., 2010). Mouse monoclonal antibodies directed against human β-actin (Sigma, Saint Louis, MO) were employed as a loading control. After primary antibody incubation, the membranes were washed 3X with TBST then incubated with either anti-rabbit or anti-mouse IgG secondary antibody conjugated to horseradish peroxidase (KPL, Gaithersburg, MD) for 1 h at room temperature, washed 3X in TBST and detected by chemiluminescense following the manufacturer's directions (ECL SuperSignal West Pico Chemiluminescent Substrate, Pierce, Rockford, IL). Visualization and digital imaging of immunoblots was performed on a FluorChem HD2 Imaging System **(**Alpha Innotech Corporation, Leandro, CA).

### Densitometry

**Figure 1:**
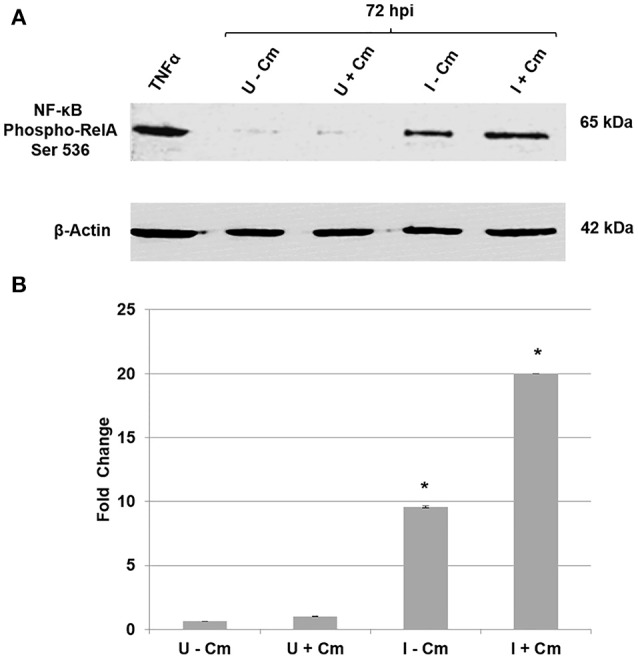
**Immunoblot analysis of ***C. burnetii*** modulation of NF-κB activation. (A)** *Top panel*—immunoblot detection of phosphorylated RelA. *Bottom panel*—β-actin loading control. Uninfected THP-1 cells without Cm (U−Cm). Uninfected THP-1 cells with Cm (U+Cm). Infected THP-1 cells without Cm (I−Cm). Infected THP-1 cells with Cm (I+Cm). All samples were collected at 72 hpi. TNF-α-treated cells were used as positive controls for RelA phosphorylation. **(B)** *Graph*—difference in RelA protein phosphorylation levels relative to normalized β-actin. The Y-axis represents fold changes in phosphorylated RelA and the X axis indicates samples. Results represent the mean of three independent experiments. Error bars represent ± SD. Statistically significant differences between U−Cm and I−Cm or I+Cm are represented as ^*^P ≤ 0.001—(Student's *t*-test).

**Figure 2:**
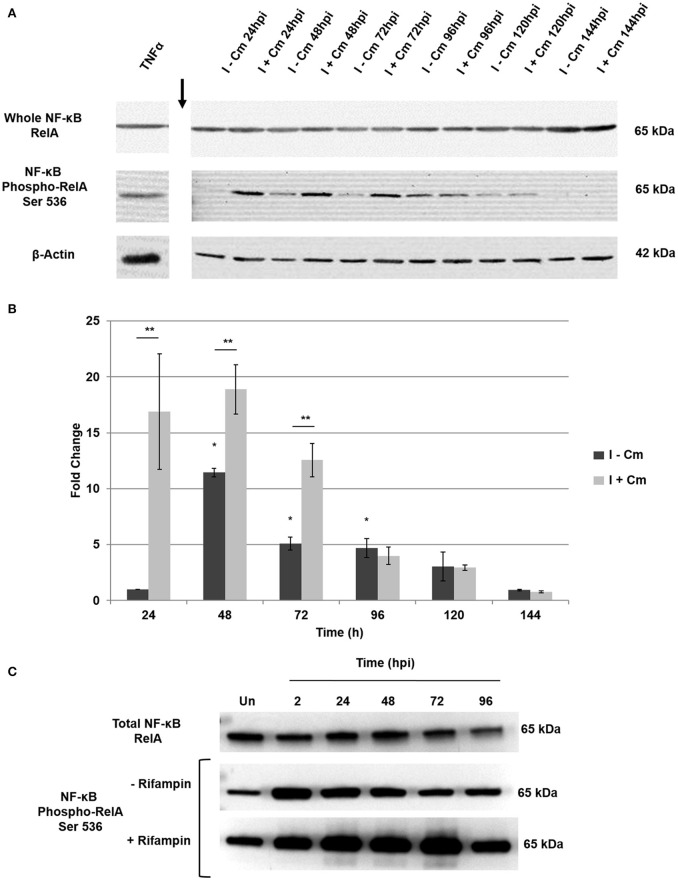
**Temporal analysis of NF-κB activation in ***C. burnetii***-infected THP-1 cells. (A)** Representative immunoblots showing NF-κB activation and controls over a time course of *C burnetii* infection. *Top panel*—RelA probed with a monoclonal antibody. *Middle panel*—phosphorylated RelA probed with a monoclonal antibody against S536. *Bottom panel*—β-actin loading control. Time (hpi) at which untreated (I−Cm) and Cm-treated (I+Cm) *C. burnetii*-infected THP-1 cells were harvested is indicated above lanes. Arrow indicates lane break. **(B)** Fold change of phosphorylated RelA vs. time in the presence and absence of Cm. Results of densitometric analysis are presented as the mean of three experiments. Error bars show ±S.E.M. Statistical differences were calculated using a *t*-test for paired samples. ^*^Signifies *P* < 0.05 of I-Cm samples compared to 24 hpi. ^**^Signifies *P* < 0.05 between paired (I−Cm to I+Cm) samples at each time point. **(C)** A representative immunoblot showing phosphorylated RelA levels in PMA-differentiated THP-1 cells treated or mock-treated with Rifampin 2—96 hpi. *Top panel*—RelA control probed with a monoclonal antibody. *Middle panel*—phosphorylated RelA from infected cells mock-treated with Rifampin (−Rifampin). *Bottom panel*—phosphorylated RelA from infected cells treated with Rifampin (+Rifampin). Time (hpi) at which cells were harvested is indicated above each lane. Un = Uninfected cells.

**Figure 3:**
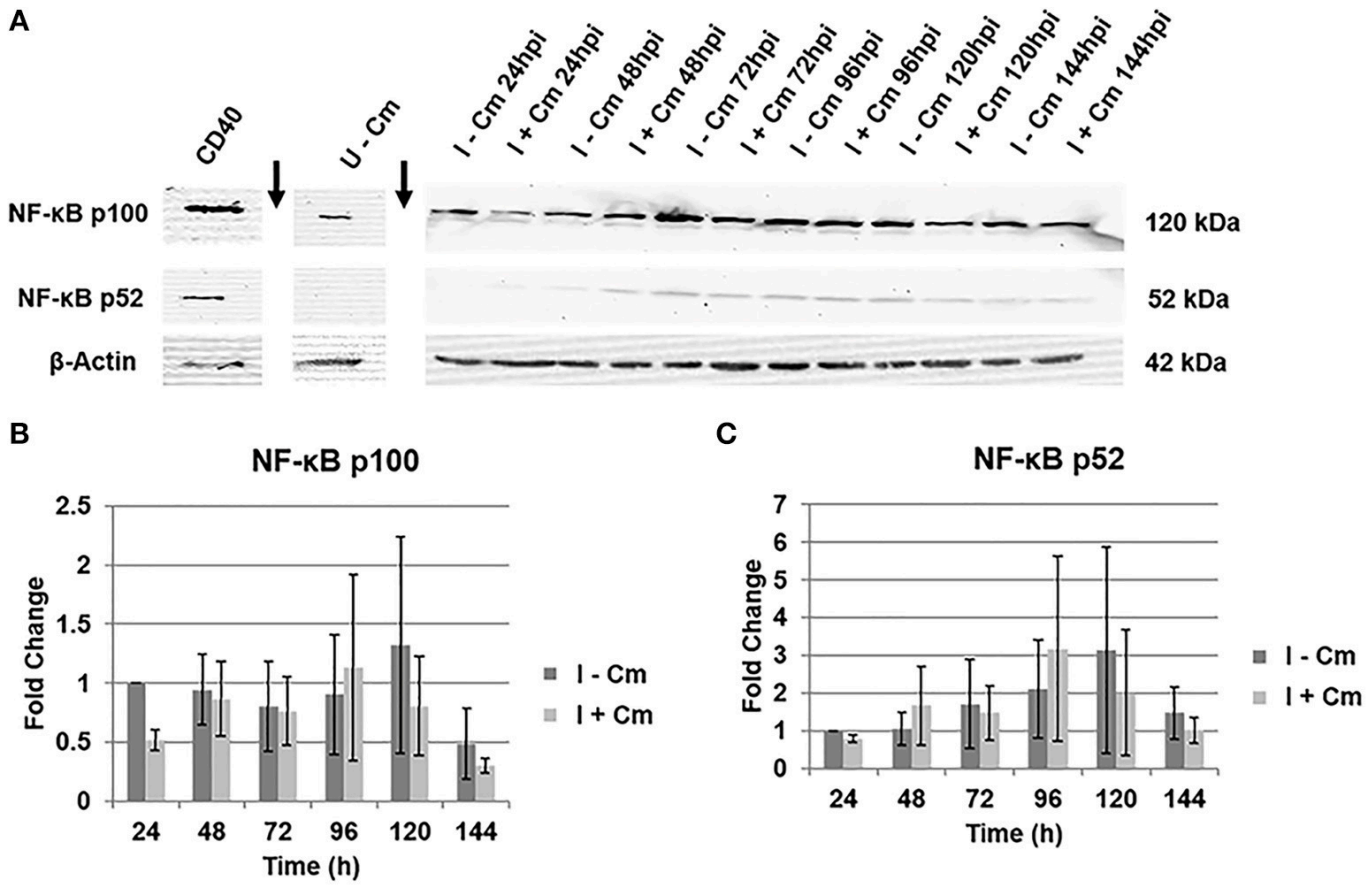
**Immunoblot analysis of NF-κB p100 and p52 in ***C. burnetii***-infected cells. (A)** Representative immunoblots showing NF-κB p100 and p52 in *C. burnetii*-infected THP-1 cells either treated with Cm (I+Cm) or left untreated (I−Cm). Blots were probed with a polyclonal rabbit antibody against NF-κB (p100/p52) of human origin (Top/Middle panels, respectively). *Bottom panel*—β-actin loading control. CD40-treated THP-1 cells served as a p52 positive control. Sample conditions and time point are indicated above each lane. Arrows indicate lane breaks. **(B)** Results of densitometric analysis showing fold changes of NF-κB p100 levels over time of infection with and without Cm after β-actin normalization. Error bars show ±S.E.M of three biological experiments. **(C)** Fold changes of NFκB p52 levels over time of infection with and without Cm after β-actin normalization. Error bars show ±S.E.M of three biological experiments.

**Figure 4:**
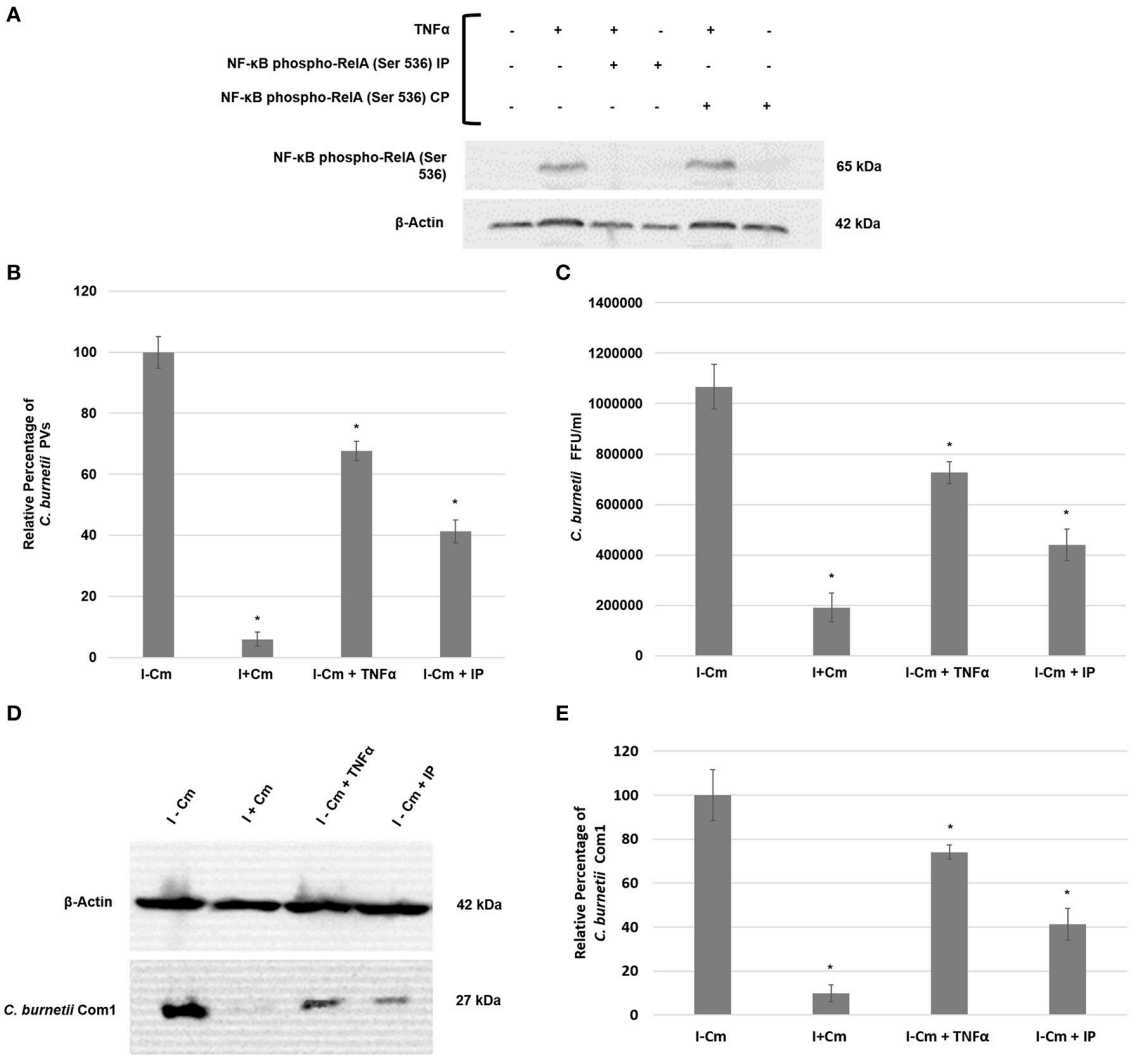
*****C. burnetii*** development following NF-κB activation or inhibition. (A)** *Top panel*—immunoblot detection of phosphorylated RelA in THP-1 cells. *Bottom panel—*β-actin loading control. Sample treatments (±) are indicated above immunoblot panels and correlate to the lanes below. **(B)** Inhibition of RelA phosphorylation in THP-1 cells impairs *C. burnetii* PV formation. 15 fields of view at 20x magnification (>100 cells/field of view) from three independent samples were analyzed for PV enumeration. Error bars show ±S.E.M. Statistical differences relative to I−Cm were calculated using a *t*-test for paired samples (^*^signifies *P* < 0.05). **(C)** Infectious progeny enumeration in Hela cells from three independent samples as measured by IFA and Fluorescent Forming Units (FFUs). Error bars show ± SEM. Statistical differences relative to I−Cm were calculated using a *t*-test for paired samples (^*^signifies *P* < 0.05). **(D)** Representative immunoblot showing Com1 levels in *C. burnetii*-infected THP-1 cells either untreated or treated with Cm, TNF-α, or RelA IP and harvested at 72 hpi. *Top panel*—β-actin control. *Bottom panel*—Com1 detected with a rabbit polyclonal antibody. **(E)** Differences in *C. burnetii* Com1 levels relative to normalized β-actin from three independent samples were analyzed. All samples were analyzed at 72 hpi. *C. burnetii* growth was examined in THP-1 cells either untreated (I−Cm), or treated with Cm (I+Cm), TNF-α, or RelA IP. Error bars represent ± SEM. Statistically significant differences relative to I–Cm are represented as ^*^P ≤ 0.05, (Student's *t*-test).

**Figure 5:**
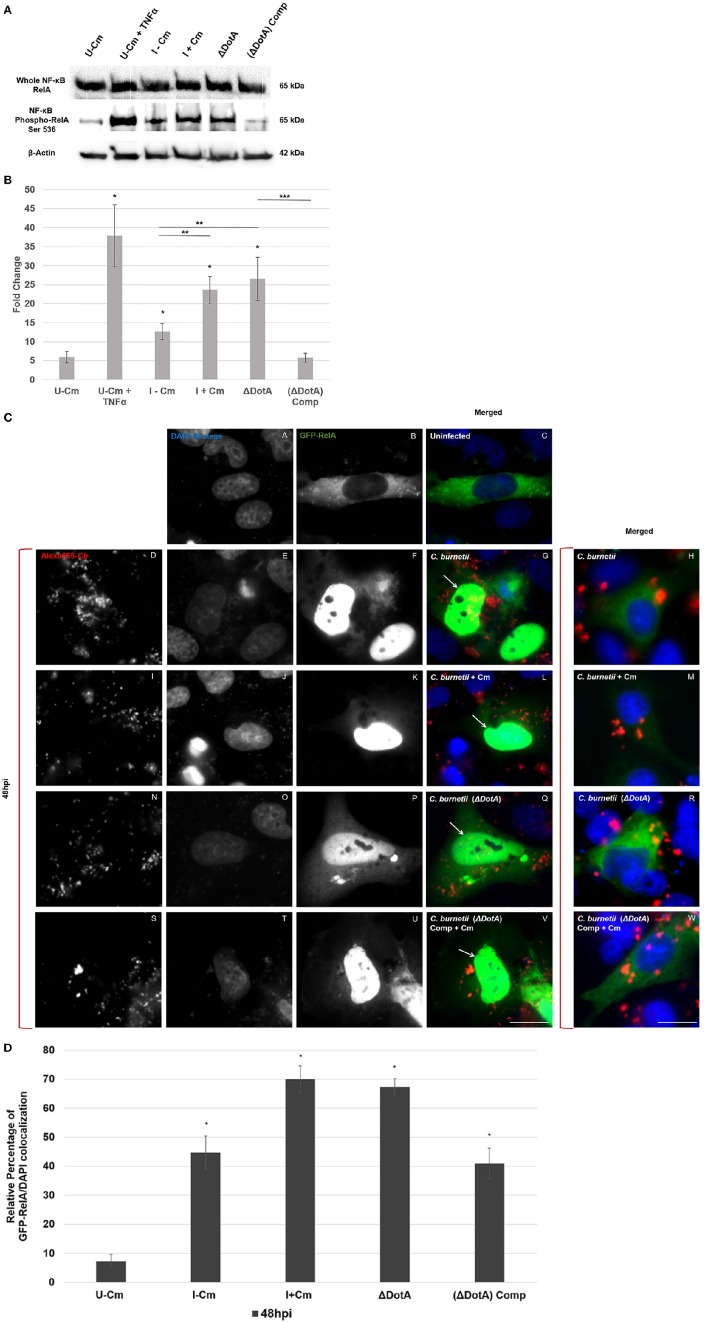
*****C. burnetii*** employs the Dot/Icm T4BSS to modulate NF-κB signaling. (A)** Immunoblot analysis of NF-κB activation in THP-1 cells (48 hpi) using wild type *C. burnetii* (± Cm), a DotA mutant (Δ*dotA*), and a *dotA*-complemented strain (Δ*dot*A) Comp. *Top panel*—detection of RelA. *Middle panel*—detection of phosphorylated RelA. *Bottom panel*—β-actin loading control. TNF-α-treated cells were used as positive RelA phosphorylation controls. **(B)** Differences in phosphorylated RelA levels relative to normalized β-actin. The Y-axis represents fold changes in phoshorylated RelA by densitometry and the X axis indicates samples. Results represent the mean of three independent experiments. Error bars represent ± SEM. For statistically significant differences, ^*^signifies *P* < 0.05 when samples are compared to U–Cm, ^**^signifies *P* < 0.05 when samples are compared between I–Cm to I+Cm or Δ*dot*A. ^***^Signifies *P* < 0.05 when samples are compared between Δ*dot*A and Δ*dot*A Comp—(Student's *t*-test). **(C)** Immunofluorescent image showing localization of GFP-RelA (green). *C. burnetii* was visualized by Alexa-555 (red), and DNA/Nuclei with Dapi (blue). HeLa cells transiently transfected with GFP-RelA (green) vector were infected with the indicated *C. burnetii* strains for 48 h. **(A–C)** Uninfected cells. **(D–G,I–L,N–Q,S–V)** Cells infected with the indicated *C. burnetii* strain in which GFP-RelA and DAPI co-localize. Arrows indicate co-localization. **(H,M,R**,**W)** Merged micrographs representing cells infected with the indicated *C. burnetii* strain where GFP-RelA did not co-localize with DAPI. Bar, 10 μm. **(D)** Quantification of GFP-RelA/DAPI co-localization. A minimum of 100 transiently transfected Hela cells from each of three separate experiments was counted to determine GFP-RelA/DAPI co-localization. Error bars show ± SD. Statistically significant differences (^*^P < 0.05, Student's *t*-test) are shown when samples are compared to U–Cm at 48 hpi.

The signal density of the detected bands in experimental samples were analyzed by ImageJ (version 1.46 h) as described previously (Schneider et al., 2012). Briefly, relative phosphorylated RelA band intensity was normalized to β-actin and quantified with respect to uninfected THP-1 cells (Figure 1A) and *C. burnetii* infected THP-1 cells at 24 hpi (Figure 2A). To examine the role of non-canonical NF-κB signaling pathway, relative NF-κB p100 and NF-κB p52 band intensity was normalized to β-actin and quantified with respect to *C. burnetii* infected THP-1 cells at 24 hpi (Figure 3). In Figure 4D, relative *C. burnetii* Com1 band intensity was normalized to β-actin and quantified with respect to *C. burnetii* infected THP-1 cells at 72 hpi. For Figure 5A, relative phosphorylated RelA band intensity was normalized to β-actin and quantified with respect to uninfected THP-1 cells.

### Immunofluorescent microscopy

An indirect immunofluorescence assay (IFA) was used to enumerate PV and infectious *C. burnetii*. For immuno-staining, methanol-fixed *C. burnetii*-infected cells were incubated 1 h with polyclonal Guinea pig anti-*C. burnetii* primary sera diluted in PBS, 2% BSA. After 3 PBS washes, samples were incubated 1 h with Alexa488-conjugated goat anti-Guinea pig secondary IgG (Molecular probes, Thermo Fisher) also diluted in PBS, 2% BSA. DAPI (4',6-diamidino-2-phenylindole) was used in the secondary incubation to stain total DNA. Labeled cells were visualized using a Nikon Eclipse TE 2000-S fluorescence microscope with a Nikon DS FI1 camera and NIS-ELEMENTS F 3.00 software (Mahapatra et al., 2010). A minimum of 15 fields per sample were counted using the 20x objective. All experiments were performed with 3 biological samples and statistical analyses were performed using a paired Student's *t*-test.

### Transfections and GFP-RelA/DAPI co-iocalization assay

Hela cells were transiently transfected with the GFP-RelA plasmid (Addgene) using X-tremeGENE 9 DNA Transfection Reagent (Roche Life Sciences), as described by the manufacturer. Transfected cells were grown overnight and then inoculated with either *C. burnetii* NMII, *C. burnetii* Δ*dotA* (Beare et al., 2012), or a *C. burnetii dotA*-complemented strain (Beare et al., 2012). Separate cells were also infected with *C. burnetii* NMII concurrent with the addition of Cm (Mahapatra et al., 2010). *C. burnetii* Δ*dotA* strain and *dotA*-complemented strains were generously provided by Dr. Robert A. Heinzen (Rocky Mountain Laboratories, NIAID, NIH, Hamilton, MT) (Beare et al., 2012). All strains were used at an MOI of 25. At 24 and 48 hpi, cells were methanol-fixed and IFA performed as above using Guinea pig anti-*C. burnetii* primary antibody and Alexa555-conjugated goat anti-Guinea pig secondary IgG (Molecular probes, Thermo Fisher). DAPI was used to stain total DNA. Subcellular co-localization of GFP-RelA and DAPI in transfected cells was visualized as above using a 60x oil objective. A minimum of 100 transfected cells were scored per sample. Data shown are the means of three independent experiments and statistical analyses were performed using a paired Student's *t*-test.

## Results

### NF-κB activation is modulated by *C. burnetii* proteins during infection

RelA is one of the most extensively studied NF-κB complex subunits (Sakurai et al., 2003; Bonizzi and Karin, 2004; Perkins, 2007). Similar to cRel and RelB, it contains a 300-amino acid region with homology to the Rel proto-oncogene (RH domain) and a transactivation domain (Sakurai et al., 2003; Bonizzi and Karin, 2004; Perkins, 2007). The RH domain harbors motifs for nuclear localization and binding to specific DNA sequences, while the transactivation domain contains phosphorylation sites that remain bound to the inhibitor IκB while in the cytoplasm (Sakurai et al., 2003; Bonizzi and Karin, 2004; Perkins, 2007). Phosphorylation of serine 536 (S536) in the transactivation domain is required for optimal activation (Sakurai et al., 2003; Bonizzi and Karin, 2004; Viatour et al., 2005; Perkins, 2007). To determine if NF-κB is modulated by *C. burnetii*, NF-κB activation was assayed in THP-1 cells via detection of RelA phosphorylation during infection and treatment with Cm (Sakurai et al., 2003). Figures 1A,B reveal that NF-κB is activated during infection as evidenced by increased levels of phosphorylated RelA, and that *C. burnetii* protein synthesis is required to modulate the level of activation. Compared to uninfected cells, RelA phosphorylated protein levels increased ~10-fold in *C. burnetii*-infected cells. However, phosphorylated RelA levels were ~20-fold higher in infected cells treated with Cm. These results indicate that *C. burnetii* proteins modulate NF-κB signaling during infection.

### *C. burnetii* modulates NF-κB activation temporally during infection

To assess the dynamics of NF-κB activation throughout infection, we examined infected THP-1 cells from 24–144 hpi. Cells were either mock-treated or transiently treated with Cm. We hypothesized that NF-κB activation would respond directly to *de novo* bacterial protein synthesis depending on the stage of infection (early [24–48 hpi], mid [48–96 hpi], or late [96–144 hpi]). To test this hypothesis, we first investigated the total levels of RelA. As shown in Figure 2A, RelA levels at each time post-infection were consistent throughout infection, indicating *C. burnetii* does not modulate the relative expression of RelA. We next assessed RelA phosphorylation in the presence and absence of transient Cm treatment. Figures 2A,B show that in the absence of transient Cm treatment, phosphorylated RelA levels remain low at 24 hpi (early infection). However, when compared to 24 hpi, RelA phosphorylation levels significantly increase at 48 hpi (early to mid-infection) and remain elevated through 96 hpi (mid infection). Notably, transient application of Cm at 0, 24, and 48 hpi resulted in even higher levels of phosphorylated RelA at 24, 48, and 72 hpi, respectively, suggesting that *C. burnetii* proteins dampen NF-κB activation during infection. Interestingly, application of Cm at 72 hpi did not alter RelA phosphorylation levels at 96 hpi relative to infected cells in the absence of Cm. During late infection times (96–144 h), RelA phosphorylation was reduced and *de novo C. burnetii* protein synthesis did not alter NF-κB activation. Combined, these results indicate that infection of THP-1 cells by *C. burnetii* involves temporal modulation of NF-κB activation via RelA phosphorylation. While *C. burnetii* infection triggers NF-κB activation, *de novo C. burnetii* proteins significantly suppress NF-κB activation during early and middle stages of intracellular growth. To confirm these findings, we examined the effect of treatment with the mRNA synthesis inhibitor rifampin on temporal NF-κB activation during infection. Additionally, to determine if these observations were infection model-specific, we assessed infection of PMA-differentiated THP-1 macrophage-like cells. Cells were treated with rifampin at the time of infection, and RelA phosphorylation assessed at 2, 24, 48, 72, and 96 hpi. Figure 2C shows that RelA phosphorylation levels in differentiated THP-1 cells infected with *C. burnetii* increased relative to uninfected cells. Furthermore, inhibition of bacterial mRNA synthesis resulted in even higher levels of RelA phosphorylation that were evident through 96 hpi similar to Figure 2A. These observations support our findings that indicate *C. burnetii* proteins temporally modulate NF-κB activation during infection.

### *C. burnetii* does not modulate NF-κB activation via the non-canonical pathway

The results above demonstrate the involvement of RelA in NF-κB activation. NF-κB transcription factors are typically activated by either the canonical or non-canonical signaling pathway (Beinke and Ley, 2004; Bonizzi and Karin, 2004; Park et al., 2005; Viatour et al., 2005; Perkins, 2007). The canonical pathway transmits signals via RelA activation, while the non-canonical pathway functions through NF-κB p52 activation (Beinke and Ley, 2004; Bonizzi and Karin, 2004; Park et al., 2005; Viatour et al., 2005; Perkins, 2007). Formation of active p52 occurs via proteolytic processing of the p100 precursor (Beinke and Ley, 2004; Bonizzi and Karin, 2004; Park et al., 2005; Viatour et al., 2005; Perkins, 2007). Therefore, to determine if the non-canonical pathway is activated during *C. burnetii* infection of THP-1 cells, we used immunoblot analysis to detect NF-κB p100/p52. Figure 3 clearly demonstrates that *C. burnetii* does not modulate host cell NF-κB p100 and p52 levels over the course of infection (24—144 hpi) in the presence or absence of bacterial protein synthesis. When compared to *C. burnetii*-infected cells at 24 hpi, NF-κB p100 levels remain constant throughout infection, and addition of Cm does not significantly impact p100 expression (Figure 3B). In contrast, p52 levels are barely detectable and do not significantly change in the presence or absence of Cm (Figure 3C). Together, these data reveal that NF-κB activation in infected THP-1 cells does not involve non-canonical NF-κB signaling.

### Inhibition of NF-κB activation impairs *C. burnetii* development

To determine if *C. burnetii*-mediated NF-κB activation is essential for intracellular survival and growth, we treated THP-1 cells with an inhibitory peptide (IP) that competitively inhibits RelA (S529/S536) phosphorylation to suppress NF-κB activation. Inhibition of RelA phosphorylation was confirmed by immunoblotting using TNF-α to trigger RelA phosphorylation (Figure 4A). Figure 4A demonstrates the effectiveness of TNFα to induce RelA phosphorylation and the IP's ability to inhibit RelA phosphorylation. To measure *C. burnetii* development following induction (TNFα) or inhibition (IP) of NF-κB activation, we analyzed (i), the number of treated THP-1 cells with PV relative to infected, untreated cells, (ii) the number of infectious progeny produced in Hela cells by treated THP-1 cell lysates relative to infected, untreated THP-1 cell lysates, and (iii) levels of the major outer membrane protein Com1 in treated and untreated THP-1 cells as a measure of C. *burnetii* total protein. Figures 4B–D show that PV formation, infectious progeny, and *C. burnetii* Com1 levels were reduced by approximately 20–30% in THP-1 cells pretreated with TNF-α. Of particular interest, Figure 4B demonstrates that, compared to *C. burnetii* infected cells, PV are reduced by approximately 60% in IP-treated cells. Figures 4C–E confirm and support these observations, demonstrating that the number of infectious *C. burnetii* produced (Figure 4C) within IP-treated cells as well as the amount of Com1 detected in these cells (Figures 4D,E) was reduced by approximately 60%. Collectively, these results demonstrate that *C. burnetii* development is significantly impaired when NF-κB activation is inhibited. Additionally, unlike Cm treatment, *C. burnetii* is able to overcome high levels of activated NF-κB induced by TNF-α application. However, their growth is marginally inhibited.

### *C. burnetii* requires a functional Dot/Icm T4BSS to modulate NF-κB signaling

Pathogenic microorganisms employ unique strategies to interfere with NF-κB signaling (Rahman and McFadden, 2011). Bacteria modulate NF-κB signaling (activation or suppression) depending on requirements of their intracellular lifestyle and niche (Rahman and McFadden, 2011). *C. burnetii*'s close relative, *L. pneumophila*, utilizes over 10 Dot/Icm effectors to induce a biphasic pattern of NF-κB activation (Rahman and McFadden, 2011; Shin, 2012). To determine if the *C. burnetii* T4BSS plays a role in modulating NF-κB, we analyzed RelA phosphorylation in THP-1 cells infected with either wild type *C. burnetii* (± Cm), a *C. burnetii* T4BSS Δ*dotA* mutant, or a *dotA*-complemented strain. Figures 5A,B reveal that, compared to cells infected with wild type *C. burnetii* (I−Cm), levels of phosphorylated RelA were higher in cells infected with *C. burnetii* (I+Cm) or the Δ*dotA* mutant. However, levels of phosphorylated RelA were similar in cells infected with either wild type *C. burnetii* (−Cm) or the Δ*dotA* complemented strain. Together, these results suggest that *C. burnetii* protein synthesis and a functional T4BSS are required for NF-κB modulation. To confirm this observation and determine if this event extends to RelA nuclear translocation, we transiently transfected Hela cells with GFP-RelA and examined nuclear translocation when infecting cells with either wild type *C. burnetii* (± Cm), the Δ*dotA* mutant, or the Δ*dotA*-complemented strain. Migration of GFP-RelA into the nucleus indicates NF-κB activation. Using immunofluorescence microscopy we visualized RelA colocalization with DAPI (DNA stain). Figure S1 and Figure 5C show that at 24 and 48 hpi, GFP-RelA localized to the nucleus regardless of the *C. burnetii* strain used to infect the cells. However, quantification of the amount of GFP-RelA in the nucleus of infected cells revealed that wild type *C. burnetii* (+Cm) and the Δ*dotA* mutant had approximately 50% more cells with nuclear-localized GFP-RelA (Figure 5D). These data clearly demonstrate that NF-κB activation levels are significantly higher in infected cells treated with Cm and cells infected with the Δ*dotA* mutant, suggesting that T4BSS effectors play a crucial role in modulating NF-κB signaling.

## Discussion

Manipulation of host NF-κB signaling via secreted effector activity is a strategy used by many microbial pathogens to thwart innate and adaptive immune responses (Rahman and McFadden, 2011). NF-κB activation typically induces the expression of hundreds of genes (Beinke and Ley, 2004; Bonizzi and Karin, 2004; Park et al., 2005; Viatour et al., 2005; Rahman and McFadden, 2006, 2011; Perkins, 2007). Genes targeted by NF-κB include those encoding pro-inflammatory cytokines, chemokines, and adhesion molecules that regulate recruitment and trafficking of immune cells to the site of infection (Beinke and Ley, 2004; Bonizzi and Karin, 2004; Park et al., 2005; Viatour et al., 2005; Perkins, 2007). NF-κB activation also induces the transcription of genes such as defensins that have direct microbicidal activity, and enzymes that generate reactive intermediates (Beinke and Ley, 2004). NF-κB acts as a major molecular link between the launch of innate and adaptive immunity by facilitating T cell activation via induction of MHC proteins and CD80/86 in antigen-presenting cells (Beinke and Ley, 2004). B cell differentiation is also usually stimulated by NF-κB activation (Beinke and Ley, 2004). Additionally, NF-κB plays a critical role in expression of anti-apoptotic proteins (e.g., c-IAP-1/2, AI/Bfl-1, Bcl-2, and Bcl-X_L_) (Beinke and Ley, 2004; Rahman and McFadden, 2011). Regulation of the cell-cycle protein cyclin D1, which increases cellular survival and proliferation, is also dependent on NF-κB activation (Beinke and Ley, 2004; Rahman and McFadden, 2011). Thus, master regulators such as NF-κB are prime targets for pathogenic microorganisms that promote survival by “regulating the regulator” to meet the requirements of their intracellular life cycle. In the current study, we demonstrate that *C. burnetii* modulates host NF-κB during infection in a T4BSS-dependent manner.

We discovered that *C. burnetii* modulates NF-κB activation through a process that requires *de novo* bacterial protein and mRNA synthesis (Figures 1, 2). Our findings clearly indicate that *C. burnetii* promotes NF-κB activation via RelA phosphorylation in a temporal manner, and bacterial protein and mRNA synthesis inhibitors alter activation. These findings indicate that *C. burnetii* maintains a balance between activation and suppression of NF-κB signaling during infection. Depending on the stage of infection, bacteria typically either activate or suppress NF-κB signaling (Rahman and McFadden, 2011). Studies on *C. burnetii*'s close phylogenetic relative reveal that *L. pneumophila* induces a biphasic pattern of NF-κB activation in human epithelial cells (Bartfeld et al., 2009; Rahman and McFadden, 2011; Shin, 2012). Short term activation at early time of infection (<8 hpi) is followed by decreased activation, which is then followed by long term induction of NF-κB later in infection (Bartfeld et al., 2009). Unlike *L. pneumophila*, our data suggest that *C. burnetii* activates NF-κB at a low level for at least the first 5 days of a ~6 day infection cycle (Figure 2A). Importantly, activation is suppressed relative to Cm- and rifampin-treated infections, indicating the response of the host cell is robust NF-κB activation in the absence of ongoing *C. burnetii* macromolecular synthesis. Activation and suppression of NF-κB signaling during early to mid-infection likely promotes cell survival and prevents a robust immune response.

NF-κB can be activated by canonical and non-canonical pathways. Our findings indicate that *C. burnetii* does not modulate NF-κB via the non-canonical pathway (Figure 3). This finding is crucial to narrow *C. burnetii* modulation of NF-κB activation to canonical pathway molecular components upstream and downstream of RelA. Canonical NF-κB pathway-associated molecular components, such as host inducer ligands, receptors, adaptor molecules, IκB proteins, and kinases, may play a crucial role in *C. burnetii*-mediated activation or suppression of NF-κB signaling and are commonly targeted by intracellular bacterial pathogens (Rahman and McFadden, 2011). Examples include *Shigella flexneri* and *Yersinia* spp. that use the type III secretion system effectors OspG and YopP/J, respectively, to prevent IκB degradation, maintaining NF-κB inactive in the cytosol (Bhavsar et al., 2007). Conversely, activation of canonical NF-κB signaling protects several intracellular pathogens including *Mycobacterium tuberculosis* (Dhiman et al., 2007), *Bartonella henselae* (Kempf et al., 2005), *Chlamydia pneumonia* (Wahl et al., 2001), *Rickettsia rickettsii* (Clifton et al., 1998), and *L. pneumophila* (Abu-Zant et al., 2007) from cell death. Our results clearly indicate that inhibition of RelA phosphorylation restricts PV formation and reduces infectious progeny production (Figure 4). It is tempting to speculate that in the absence of some level of NF-κB activation, THP-1 cells prevent *C. burnetii* development by promoting pro-apoptotic mechanisms. It is likely that NF-κB-mediated anti-apoptotic genes such as *c-iap2* and *a1/bfl*-1, which are up-regulated in *C. burnetii*-infected cells (Voth et al., 2007), would not be expressed when RelA phosphorylation is abrogated. Together, these findings suggest that two opposing effects of NF-κB activation occur in *C. burnetii*-infected cells: (1) some level of NF-κB activation is required to suppress pro-apoptotic pathways, which is beneficial for the pathogen, while (2) robust NF-κB activation would induce the expression of pro-inflammatory cytokines, ultimately leading to pathogen clearance from the host.

Finally, to identify *C. burnetii* factors that modulate NF-κB signaling during infection, we investigated whether the *C. burnetii* T4BSS was involved. In a series of experiments (Figures 5A–D and Figures S1A,B) we demonstrate that bacteria lacking a functional T4BSS do not modulate NF-κB activation during infection. This suggests that *C. burnetii* uses T4BSS effectors to either directly or indirectly modulate host NF-κB activity. Multiple studies indicate that *C. burnetii* T4BSS effectors include proteins with eukaryotic-like domains, including ankyrin repeat domains, tetratricopeptide repeats, coiled-coil domains, leucine-rich repeats, GTPase domains, ubiquitination-related motifs, and multiple kinases and phosphatases (Pan et al., 2008; Voth et al., 2009; Chen et al., 2010; Toman et al., 2012; van Schaik et al., 2013). Importantly, a significant number of *C. burnetii* T4BSS effectors are translocated into the host cell cytoplasm when expressed in a surrogate *L. pneumophila* system (Pan et al., 2008; Voth et al., 2009; Chen et al., 2010; Toman et al., 2012; van Schaik et al., 2013). Distinct T4BSS effectors associate with the PV membrane, microtubules, mitochondria, and the nucleus (Pan et al., 2008; Voth et al., 2009; Chen et al., 2010; Toman et al., 2012; Larson et al., 2013, 2015; Weber et al., 2013; van Schaik et al., 2013: Weber et al., 2016). However, the specific function of the vast majority of confirmed and putative *C. burnetii* T4BSS effectors remains unknown. Interestingly, *C. burnetii*'s close phylogenetic relative, *L. pneumophila*, regulates host NF-κB activation using more than 10 Dot/Icm-dependent effectors (Rahman and McFadden, 2011; Shin, 2012). As NF-κB is one of the principal regulators of the host immune response, identifying and characterizing *C. burnetii* T4BSS effectors that modulate this pathway and thoroughly characterizing the mechanism of NF-κB modulation will significantly contribute to our understanding of *C. burnetii* pathogenic mechanisms.

## Author contributions

Conceived and designed the experiments: SM, BG, SS, JG, DV, ES. Performed the experiments: SM, BG, SS, JG. Analyzed the data: SM, BG, JG, DV, ES. Wrote the paper: SM, ES.

## Funding

This work was supported by NIH grant R15 AI072710 (to ES), NIH grant R01 AI087669 (to DV), and the Arkansas Biosciences Institute (to DV).

### Conflict of interest statement

The authors declare that the research was conducted in the absence of any commercial or financial relationships that could be construed as a potential conflict of interest.

## Supplementary material

Figure S1 *****C. burnetii*** uses Dot/Icm T4BSS to modulate NF-κB signaling. (A)** Immunofluorescent image showing localization of GFP-RelA (green). *C. burnetii* was visualized by Alexa-555 (red), and DNA/Nuclei with Dapi (blue). HeLa cells transiently transfected with GFP-RelA (green) vector were infected with the indicated *C. burnetii* strains for 24 h. **(A–C)** Uninfected cells. **(D–G,I–L,N–Q,S–V)** Cells infected with the indicated *C. burnetii* strain in which GFP-RelA and DAPI co-localize. Arrows indicate co-localization. **(H,M,R**,**W)** Merged micrographs representing cells infected with the indicated *C. burnetii* strain where GFP-RelA did not co-localize with DAPI. Bar, 10 μm. **(B)** Quantification of GFP-RelA/DAPI co-localization. A minimum of 100 transiently transfected Hela cells from each of three separate experiments was counted to determine GFP-RelA/DAPI co-localization. Error bars show ± SD. Statistically significant differences (^*^*P* < 0.05, Student's *t*-test) are shown when samples are compared to U–Cm at 24 hpi.
