# Supplementary figures and images for: Coxiella burnetii Employs the Dot/Icm Type IV Secretion System to Modulate Host NF-κB/RelA Activation
Source: Front Cell Infect Microbiol. 2016 Dec 19;6:188. doi: 10.3389/fcimb.2016.00188 (PMC5165255; doi:10.3389/fcimb.2016.00188)

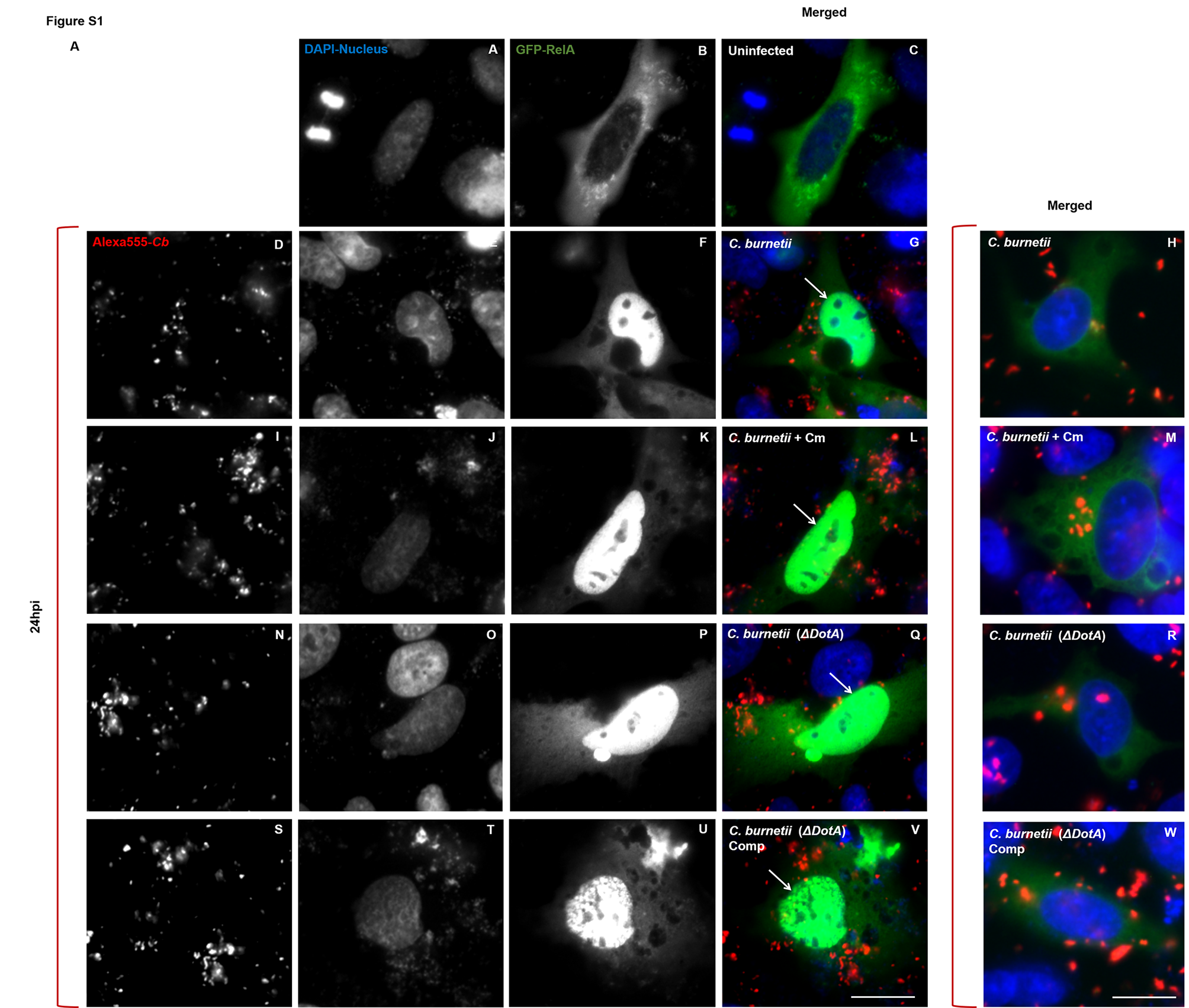

Supplement: Figure S1 — C. burnetii uses Dot/Icm T4BSS to modulate NF-κB signaling. (A) Immunofluorescent image showing localization of GFP-RelA (green). C. burnetii was visualized by Alexa-555 (red), and DNA/Nuclei with Dapi (blue). HeLa cells transiently transfected with GFP-RelA (green) vector were infected with the indicated C. burnetii strains for 24 h. (A–C) Uninfected cells. (D–G,I–L,N–Q,S–V) Cells infected with the indicated C. burnetii strain in which GFP-RelA and DAPI co-localize. Arrows indicate co-localization. (H,M,R,W) Merged micrographs representing cells infected with the indicated C. burnetii strain where GFP-RelA did not co-localize with DAPI. Bar, 10 μm. (B) Quantification of GFP-RelA/DAPI co-localization. A minimum of 100 transiently transfected Hela cells from each of three separate experiments was counted to determine GFP-RelA/DAPI co-localization. Error bars show ± SD. Statistically significant differences (*P < 0.05, Student's t-test) are shown when samples are compared to U–Cm at 24 hpi. [file Image1.TIF]

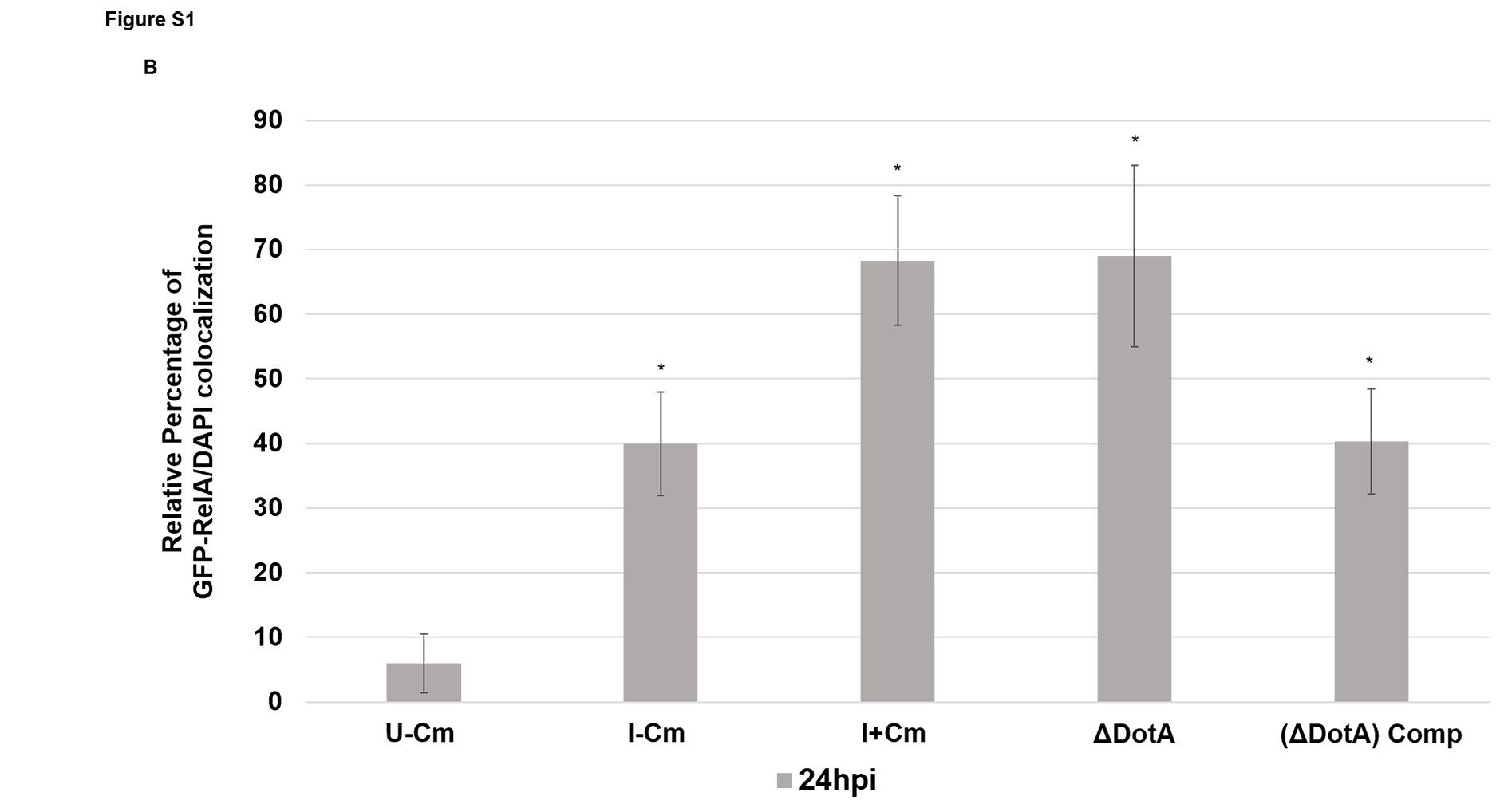

Supplement: Supplementary file 2 [file Image2.TIF]
